# Combined training in hypoxic environments improves cardiometabolic health in older adults: a systematic review and meta-analysis of randomized controlled trials

**DOI:** 10.3389/fmed.2025.1728637

**Published:** 2025-12-03

**Authors:** Hao Chen, Peng Liu, Haibo Cai, Yidi Deng, Pu Liang, Xin Jiang

**Affiliations:** 1 Department of Physical Education, Dalian University of Finance and Economics, Dalian, China; 2 College of Physical Education, Dalian University, Dalian, China

**Keywords:** cardiometabolic health, hypoxic exercise, combined exercise, older adults, body composition

## Abstract

**Objective:**

The present study was designed to investigate the effects of exercise training under hypoxic versus normoxic conditions on cardiometabolic health and body composition in older adults.

**Method:**

A systematic search was carried out in five databases, namely PubMed, Web of Science, Embase, Scopus, and the Cochrane Library. Meta-analysis, Cochrane assessment, subgroup analysis, and publication bias assessment were conducted using the Stata 18 and RevMan 5.4 analysis tools.

**Results:**

A total of 12 randomized controlled studies were included, involving 358 participants. Subgroup analysis indicated that hypoxic training (HT) could significantly enhance the cardiorespiratory fitness (CRF) of non-healthy older adults (Hedges’ g = 0.57, *p* < 0.05). The combination of hypoxia and combined training (CT) could significantly improve the CRF of older adults (Hedges’ g = 0.88, *p* < 0.05) and could reduce the systolic blood pressure (SBP) (Hedges’ g = −0.51, *p* < 0.05) and diastolic blood pressure (DBP) (Hedges’ g = −0.50, *p* < 0.05) of older adults. Regarding body composition, no significant differences were observed in body mass (BM), body mass index (BMI), fat-free mass (FFM), and fat mass (FM) for HT.

**Conclusion:**

Compared with exercise in a normoxic environment, CT (aerobic and resistance training) in a hypoxic environment better improves cardiometabolic health in older adults. Moreover, hypoxic training can enhance CRF in older adults with multiple comorbidities or diabetes, playing a role in preventing and improving cardiometabolic health in this population.

**Systematic review registration:**

https://www.crd.york.ac.uk/PROSPERO/view/CRD420251011076.

## 1 Introduction

Worldwide, the number and proportion of older individuals are on the rise (1). Among individuals aged 60–79, the prevalence of cardiovascular disease (CVD) ranges from 75 to 77% (2). CVD is the leading cause of death, accounting for 40% of deaths among seniors in this age group (3). Ageing is a degenerative process characterized by a gradual loss of bone and muscle mass, a continuous decline in function, a weakening of physiological elasticity, and an increase in fragility (4). There is a significant trend of increasing mortality in patients with advancing age (5). In fact, cardiovascular health is a stronger predictor of mortality than other identified factors such as hypertension and diabetes (6). It has also been demonstrated that CVD is associated with elevated blood pressure and reduced muscle mass (7, 8). In older adults, body mass (BM) and body mass index (BMI) may remain constant, yet visceral fat may increase while muscle mass and bone metabolic mass may decrease (9).

Exercise is recognized as a potent non-pharmacological therapeutic intervention, having been demonstrated to treat and prevent a broad spectrum of diseases (10). In older adults, exercise enhances body composition (11, 12), reduces blood pressure (13), and improves cardiometabolic health (6, 14). A strong negative correlation exists between exercise and both all-cause and CVD mortality (15). Cardiorespiratory fitness (CRF) is a robust predictor of CVD and mortality, with VO₂max serving as a classic indicator of CRF (16, 17). Blood pressure is an essential metric for evaluating cardiovascular system health and gauging the heart’s functional status (8). Research has indicated that regular exercise not only bolsters cardiovascular health and cardiometabolic function (10, 14) but also elicits anabolic processes (18), thereby reducing mortality and enhancing quality of life (19). For instance, high-intensity interval training (HIIT) can decrease patient body weight and improve cardiometabolic health (20, 21). Improvements in resting blood pressure and positive impacts on cardiometabolic health achieved through resistance training (RT) may represent an effective non-pharmacologic treatment strategy for preventing and managing elevated cardiometabolic risk in older adults, as well as for addressing hypertension (22). Previous research indicates that combined training (CT) not only elevates training intensity more effectively (23), but also demonstrates superior efficacy in reducing both systolic blood pressure (SBP) and diastolic blood pressure (DBP). Furthermore, it represents a particularly relevant non-pharmacological strategy for older adults, a population with a high prevalence of hypertension (24, 25).

The hypoxia—inducible factor and its signaling pathway represent a novel direction in the treatment of various chronic diseases (26). Intermittent hypoxia (IH) is an effective non-pharmacological intervention for managing a diverse range of diseases (27). IH promotes weight loss in both healthy and pathological individuals and exerts positive modulatory effects on cardiovascular and metabolic functions, as well as on cognitive function (28). IH can initiate the hypoxic adaptive response, which reduces the risk of future hypoxic or ischemic injuries, enhances cellular resilience and function, and maintains cellular homeostasis (27). Moreover, research has demonstrated that training under hypoxic conditions is both effective and safe (29), and can enhance physical fitness (30, 31). Hypoxic training (HT) has been shown to effectively increase maximal oxygen uptake (VO₂max) in athletes, thereby improving their performance (32, 33). HT has also proven effective in improving body composition and cardiometabolic health in overweight or obese individuals (34, 35). Additionally, HT has been associated with significant improvements in cognitive function in older adults (36, 37). However, existing evidence indicates that HT has a positive impact on improving cardiometabolic health and body composition in older adults (38–40). Intermittent hypoxic training elicits a coordinated physiological cascade primarily mediated by the upregulation of hypoxia-inducible factor-1 (HIF-1). As the key oxygen-sensitive regulator, HIF-1 activation initiates multifaceted adaptations: it simultaneously enhances mitochondrial biogenesis and fat oxidation while modulating leptin levels, processes synergistically reinforced by hypoxia-induced appetite suppression that collectively reduce energy intake and elevate metabolic rate (41–43). In parallel, HIF-1 drives cardiovascular improvements through upregulated vascular endothelial growth factor (VEGF) expression, resulting in arteriolar dilation and enhanced capillary function that collectively ameliorate hypertension (44). Furthermore, this regulatory framework stimulates erythropoiesis via erythropoietin (EPO) production, ensuring adequate iron utilization for hemoglobin synthesis and erythrocyte maturation (45). These integrated metabolic, vascular, and hematological adaptations demonstrate the systemic efficacy of hypoxic training in promoting physiological resilience and collectively contribute to improved cardiometabolic health.

Currently, the superiority of HT over normoxic training (NT) with respect to cardiometabolic health and body composition in older adults remains ambiguous. Therefore, it is imperative to conduct systematic evaluations and meta-analyses to assess the advantages of HT in promoting cardiometabolic health and optimizing body composition among older adults.

## 2 Materials and methods

This paper adheres to the guidelines of the Preferred Reporting Items for Systematic Reviews and Meta-Analyses (PRISMA) (46). The registration number in PROSPERO is CRD420251011076.

### 2.1 Inclusion and exclusion criteria

The inclusion criteria were based on the PICOS principle: (1) The subjects were older adults individuals, with an age of ≥60 years. This included both healthy and unhealthy older adults. Unhealthy older adults were those clearly identified in the literature as individuals with multiple comorbidities or diabetes mellitus; (2) The experimental group underwent training in either a hypobaric or normobaric hypoxic environment, while the control group received training under normoxic conditions; (3) The hypoxic environment was defined as having a fraction of inspired oxygen (FiO₂) ≤ 17.4% or an altitude ≥ 1,500 meters; (4) Only randomized controlled trials (RCTs) were eligible for inclusion; and (5) The primary outcome indicators were related to cardiometabolic health, encompassing CRF, SBP, and DBP. The secondary outcome indicator was body composition, which included BM, BMI, fat-free mass (FFM), and fat mass (FM).

The exclusion criteria were as follows: (1) Studies that were not relevant to the subject matter; (2) Review articles, systematic reviews, and meta-analyses; (3) Publications where the full text did not include information regarding the outcome indicators; (4) Studies involving participants who were younger than 60 years of age; (5) Studies that only involved hypoxic exposure without the relevant training components (as per the study’s focus on training under hypoxic conditions); (6) Unpublished studies/unpublished data; and (7) non-English language studies. In the conduct of this review, only English-language studies were included. Non-English studies, even if they held valuable information, were excluded on account of the challenges in translation and ensuring correct interpretation. Failure to address these challenges properly could lead to biases in the research outcomes.

### 2.2 Literature search

The literature search was conducted in databases including PubMed, Web of Science, Embase, Scopus, and the Cochrane Library. The search period spanned from the database inception dates to March 5, 2025. Relevant search terms were combined using Boolean logic operators (AND, OR) to optimize the search strategy. The detailed search strategies and results are presented in Supplementary Table 1. In addition, the references of the included studies and meta-analyses were manually screened in this paper to minimize the potential for overlooking relevant studies.

### 2.3 Literature screening

Literature searches were carried out in various databases following the pre-determined search strategies. The retrieved literature was then imported into EndNote X9 software to eliminate duplicate entries. Subsequently, the titles of the literature pieces were reviewed to exclude those that were irrelevant to the study’s scope. In addition, the abstracts or full texts of the remaining literature were examined in accordance with the inclusion and exclusion criteria to further screen out ineligible studies.

### 2.4 Data extraction

The primary data extracted from the included studies comprised the following: first author, publication year, experimental groups, age and gender of participants, sample size, FiO₂/altitude, intervention protocol, load intensity, training duration, training frequency, and outcome measures.

The mean, standard deviation, and sample size of each outcome indicator were extracted for pre-intervention versus post-intervention. In studies where standard errors (SEM) were reported, the standard deviation (SD) was calculated using the equation SD = SEM × $\sqrt{N}$, where SD represents the standard deviation, SEM denotes the standard error of the mean, and *N* refers to the sample size. Subsequently, the standard deviation of the mean difference was calculated as follows:


$$SD_{\text{diff}}=\sqrt{SD_{\mathrm{pre}}^{2}+SD_{\text{post}}^{2}-2r\times SD_{\mathrm{pre}}\times SD_{\text{post}}}$$

Here, SD*_diff_* represents the standard deviation of the differences between pre- and post-intervention values. SD*_pre_* and SD*_post_* stand for the standard deviations of the pre-intervention and post-intervention measures respectively, while r denotes the correlation coefficient between the pre- and post-intervention measurements. When the correlation is not reported, the within—participant correlation coefficient *r* is defaulted to 0.5.

A sensitivity analysis was performed to evaluate the impact of varying correlation coefficients (r) ranging from 0.5 to 0.9 on the pooled outcome estimates (Supplementary Table 2). The direction, magnitude, and confidence intervals of the pooled effect sizes remained largely consistent across the different *r* values, indicating that the results were insensitive to the chosen *r* value and that the conclusions are robust.

### 2.5 Risk of bias assessment for included studies

The Cochrane Collaboration RCT bias evaluation tool in Revman 5.4 software was employed to assess: (1) Random sequence generation; (2) Allocation concealment; (3) Blinding of participants and personnel; (4) Blinding of outcome assessment; (5) Incomplete outcome data; (6) Selective reporting; and (7) Other bias.

### 2.6 GRADE analysis

The Grading of Recommendations Assessment, Development, and Evaluation (GRADE) method was utilized to evaluate the quality of the evidence. The GRADE approach assesses the certainty of the evidence, classifying it into one of four categories: very low, low, moderate, or high.

### 2.7 Statistical analysis

Revman 5.4 software was employed to generate risk-of-bias assessment maps for the included studies. Meta-analysis and the creation of forest plots for the outcome indicators derived from the included literature were carried out using Stata 18 software and Graphpad Prism 8 software. The data on the outcome indicators reported in the literature were continuous variables. Hedges’ g was selected as the effect indicator. Exact calculations were applied to determine the bias correction factors, and the effect sizes were computed using Hedges’ g along with Olkin’s corrected standard errors.

The heterogeneity of the outcome indicators was analyzed by means of *I*^2^ values and *p*-values. When the *I*^2^ value was less than 50% and the *p*-value was greater than 0.1, it signified low heterogeneity among the findings across different studies. In such cases, the fixed-effects model with inverse variance was utilized for the analysis. Conversely, when these conditions were not met, it indicated higher heterogeneity of the results across studies, and the random-effects model with the DerSimonian-Laird method was employed for the analysis. A *p*-value less than 0.05 was indicative of a statistically significant difference.

Subgroup analyses of the key outcome indicators were conducted, with the health status of older adults and different types of exercise being used as subgroups. The effect sizes were categorized as negligible (0.2), small (0.2–0.5), medium (0.5–0.8), and large (> 0.8).

Publication bias for each outcome indicator was evaluated through the Egger regression test. If the *p*-value obtained from the test was less than 0.05, it was concluded that there was significant publication bias. On the other hand, a *p*-value greater than or equal to 0.05 indicated the absence of significant publication bias.

## 3 Results

### 3.1 Literature search process and screening results

**Figure 1:**
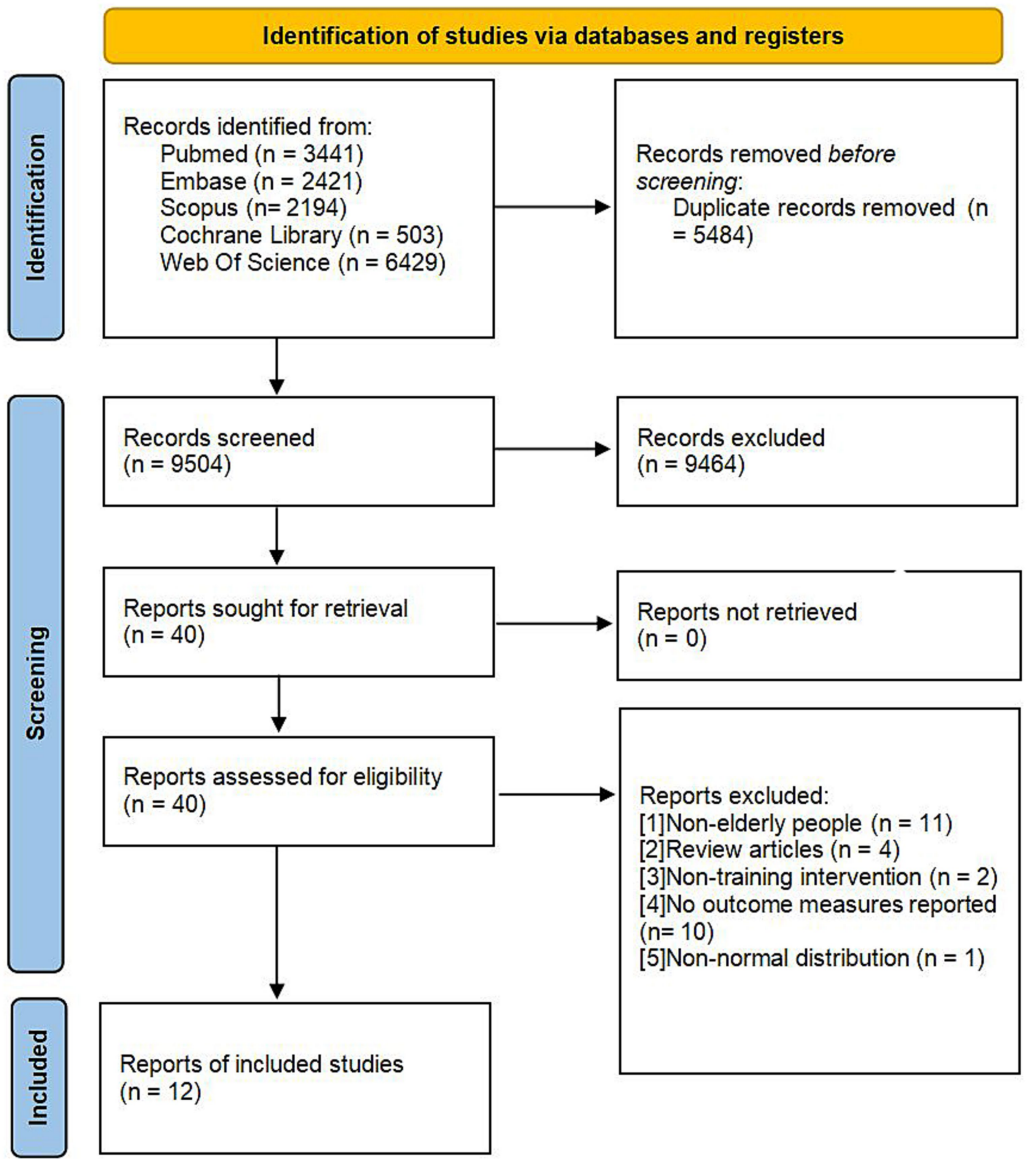
PRISMA flowchart of study selection.

A total of 14,988 articles were retrieved from the databases. After eliminating 5,484 duplicate studies and excluding 9,464 studies that were irrelevant to the research topic, the full texts of the remaining 40 articles were thoroughly examined. Ultimately, 12 studies fulfilled the established inclusion criteria and were incorporated into the analysis. The flowchart depicting the literature screening process is illustrated in Figure 1, and the fundamental details of the included studies are presented in Table 1.

**Table 1:** Main characteristics of studies included in the meta-analysis.

Study	Basic information			Intervention program			Outcome
	Participants (M/F)	Age (M ± SD)/year	BMI (M ± SD)/kg/m^2^	Duration/Frequency	FiO_2_/Time	Modality/Exercise intensity	
Allsopp, GL 2020 (40)	HT: 6/4 NT: 6/4	HT: 65.9 ± 1.1 NT: 64 ± 0.8	HT: 24.9 ± 1.1 NT: 23.9 ± 0.8	8 weeks 2 days/wk.	14.4% 60 min	Resistance training 70% 1RM	BM (NS), BMI (NS), CRF (NS), SBP (NS), DBP↓
Park, H-Y 2019 (54)	HT: 0/12 NT: 0/12	HT: 66.50 ± 0.90 NT: 66.50 ± 0.67	HT: 26.00 ± 0.61 NT: 25.63 ± 0.35	12 weeks 3 days/wk.	14.5% 90–120 min	Resistance training and aerobic training 60–70%HRmax	BM↓, FFM↑
Pramsohler, S 2017 (47)	HT: 7/12 NT: 5/11	HT: 80.2 ± 7.2 NT: 82 ± 7.8	HT: 25.2 ± 5.3 NT: 25.8 ± 6.2	3 weeks 7 training sessions	15.27% 30 min	Aerobic training 80% VO_2_peak	BM (NS), BMI (NS), CRF (NS), SBP (NS), DBP (NS)
Timon, R 2021 (56)	HT: 17 NT: 18	HT: 68.46 ± 3.8 NT: 70.35 ± 3.3	HT: 26.4 ± 3.3 NT: 27.1 ± 3.9	24 weeks 3 days/wk.	16.1% 45 min	Resistance training 60–80% VO_2_max	FFM (NS), FM (NS)
Törpel, A 2020 (51)	HT: 9/10 NT: 9/8	HT: 68.1 ± 4.6 NT: 67.8 ± 4.1	HT: 27.6 ± 4.2 NT: 26.9 ± 3.6	5 weeks 4 days/wk.	60 min	Resistance training SpO_2_: 80–85%	FFM (NS), FM (NS), CRF↑
Hein, M 2021 (48)	HT: 12 NT: 13	HT: 60.4 ± 5.0 NT: 63.8 ± 5.8	HT: 28.6 ± 3.0 NT: 28.4 ± 1.9	8 weeks 3 days/wk.	15% 30 min–40 min	Aerobic training 60%–70 VO_2_peak	CRF↑, SBP (NS), DBP (NS)
Chobanyan - Jürgens, K 2019 (52)	HT: 7/7 NT: 7/8	HT: 60.4 ± 5.1 NT: 63.8 ± 5.8	HT: 28.6 ± 3.0 NT: 28.3 ± 1.9	8 weeks 3 days/wk.	15% 30–40 min	Aerobic training 60–70% VO_2_max	CRF (NS)
Park, W 2024 (49)	HT: 0/12 NT: 0/12	HT: 67.83 ± 1.03 NT: 68.08 ± 0.90	HT: 27.31 ± 0.66 NT: 26.87 ± 0.38	12 weeks 3 days/wk.	14.5% 120 min	Resistance training and aerobic training 60–70%HRmax	BM (NS), BMI (NS), FFM (NS), SBP↓, DBP↓
Timón, R 2024 (55)	HT: 5/7 NT: 6/7	HT: 69.9 ± 4.7 NT: 69.6 ± 4.3	HT: 26.2 ± 1.3 NT: 25.8 ± 2.9	20 weeks 3 days/wk.	16.1% 30 min	Vibration training Vibration stimulus (18–22) Acceleration (2.6–3.8 g) Distant position form the axis of rotation (4 mm peak-to-peak)	BM (NS), BMI (NS), FFM↓, FM (NS)
Kindlovits, R 2024 (53)	HT: 14 NT: 14	72.2 ± 4.0	29.0 ± 3.8	8 weeks 3 days/wk.	3,000 m 60 min	Aerobic exercise 75% HRR	BM (NS), BMI (NS), FFM (NS), FM (NS), CRF↑
Kindlovits, R 2025a (50)	HT: 8/6 NT: 7/7	HT: 71.6 ± 3.8 NT: 74.4 ± 3.6	HT: 28.3 ± 4.0 NT: 29.4 ± 4.1	8 weeks 3 days/wk.	3,000 m 60 min	Aerobic training and resistance training 75% HRR	SBP (NS), DBP (NS)
Kindlovits, R 2025b (50)	HT: 9/5 NT: 7/7	HT:70.7 ± 4.0 NT:74.4 ± 3.6	HT: 29.3 ± 3.4 NT: 29.4 ± 4.1				
Marta Camacho - Cardenosa 2019 (57)	HT: 10 NT: 11	HT: 73.5 ± 4.7 NT: 70.2 ± 6.4	HT: 28.9 ± 4.2 NT: 29.5 ± 4.8	18 weeks 2 days/wk.	16% 25 min	Vibration training Vibration stimulus (12.6 Hz) Acceleration (2.55 g force) Distant position form the axis of rotation (4 mm peak-to-peak)	FFM (NS)

HT, hypoxic training; NT, normoxic training; M, male; F, female; wk., weeks; HRmax, max heart rate; VO_2_max, maximal oxygen consumption; CRF, cardiorespiratory fitness, including VO_2max_/VO_2peak_; SBP, systolic blood pressure; DBP, diastolic blood pressure; BM, body mass; BMI, body mass index; FFM, fat free mass; FM, fat mass.

### 3.2 Study characteristics

A total of 358 older individuals were included in the study, with an average age of 68.54 ± 5.38 years. Among them, 179 participants were assigned to the HT group and 179 to the NT group. In terms of gender, two studies had only female subjects, eight studies had both male and female subjects, and two studies did not specify the gender of the participants.

The fractional inspired FiO₂ range of the hypoxic conditions included in the study was from 16.1 to 14.4%. Specifically, nine studies focused on healthy older adults, one study involved patients with multiple comorbidities, and two studies included individuals with type 2 diabetes mellitus (T2DM).

The interventions employed in the studies were diverse, encompassing RT (*n* = 3), aerobic training (AT) (*n* = 4), CT (*n* = 3), and vibration training (*n* = 2). Regarding the outcome measures, seven studies reported data on CRF and BM. Six studies provided information on SBP, DBP, and BMI. Eight studies reported on FFM, and five studies reported on FM.

### 3.3 Risk of bias assessment results

**Figure 2:**
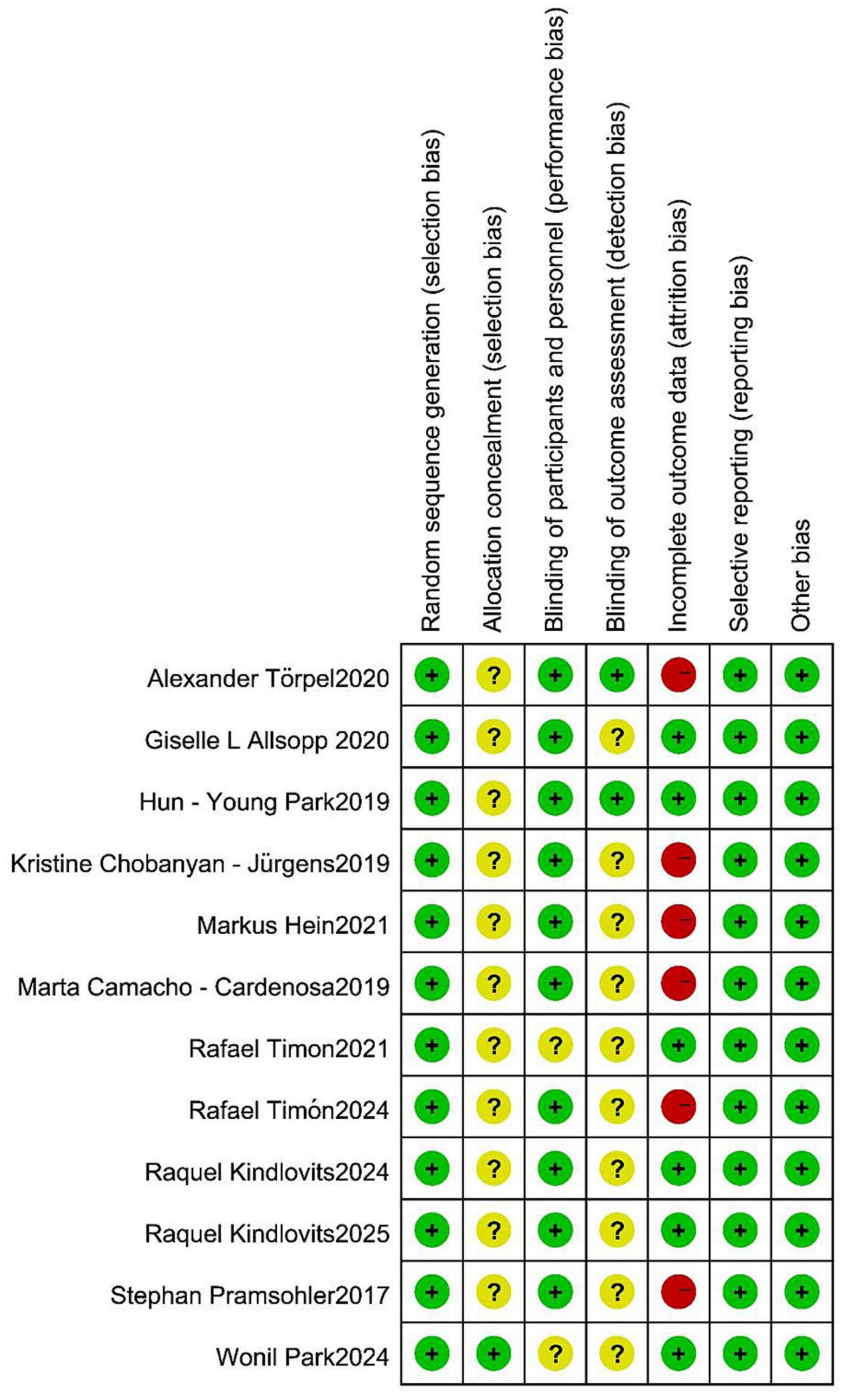
The risk assessment of bias.

The risk-of-bias assessment for the included studies is depicted in Figure 2. Only one study explicitly described the method of allocation concealment. Eight studies implemented a single-blind design, while two studies adopted a double-blind design. In all of the included studies, the participants were informed about the entire trial and its associated risks. However, six studies encountered issues related to participants dropping out of the trial during the intervention period.

### 3.4 Meta-analysis, certainty of evidence and publication bias detection

#### 3.4.1 Primary outcome indicators

**Figure 3:**
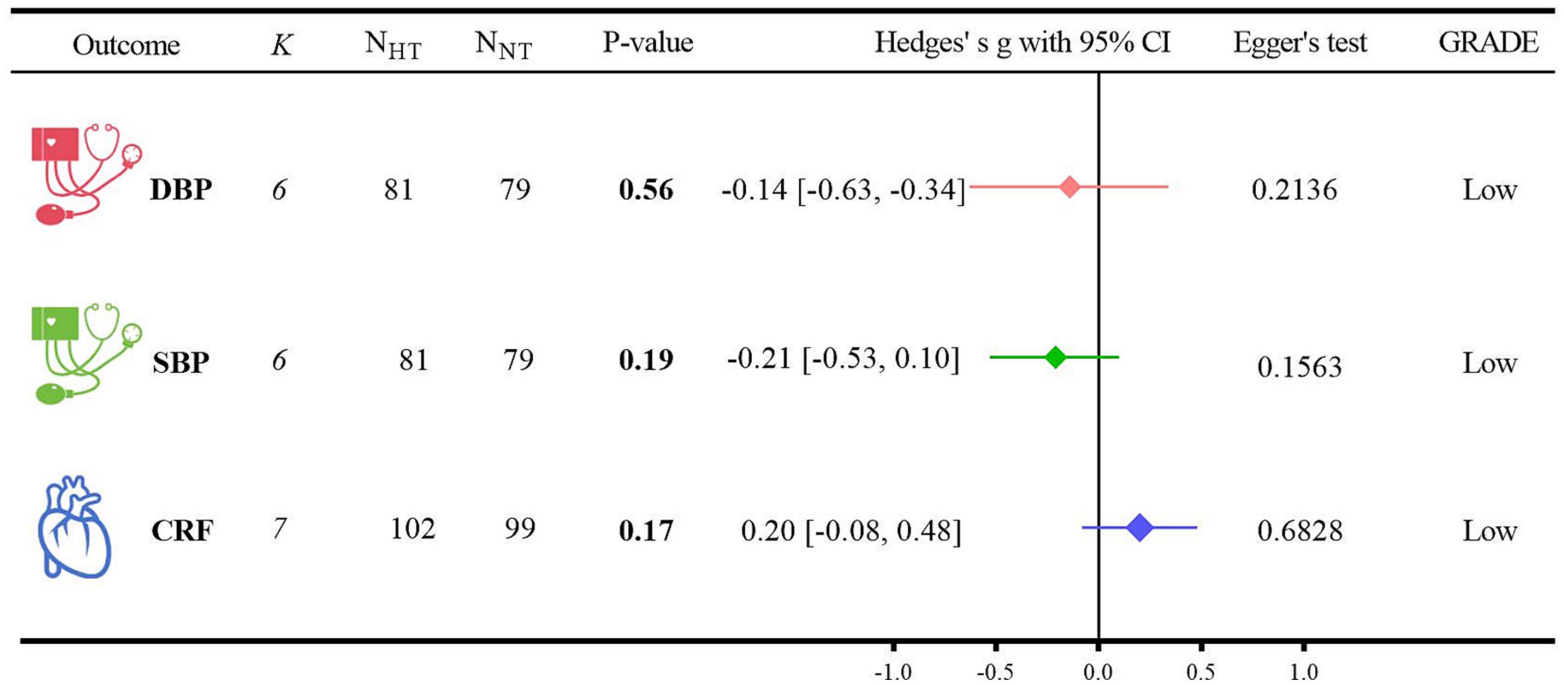
Analysis of outcome indicators of cardiometabolic health.

The analysis of the outcome indicators related to cardiometabolic health is shown in Figure 3.

(1) For the DBP analysis, five randomized controlled trials were included (40, 47–50). When compared with NT, HT did not show a significant difference from NT (Hedges’ g = −0.14, 95% CI: −0.63 to −0.34; *p* = 0.56, *I*^2^ = 57.07%). For the SBP analysis, five randomized controlled trials were also included (40, 47–50). In comparison with NT, there was no significant difference between HT and NT (Hedges’ g = −0.21, 95% CI: −0.53 to 0.10; *p* = 0.19, *I*^2^ = 0.47%). Regarding the CRF analysis, six randomized controlled trials were incorporated (40, 47, 48, 51–53). When contrasted with NT, HT did not exhibit a significant difference from NT (Hedges’ g = 0.20, 95% CI: −0.08 to 0.48; *p* = 0.17, *I*^2^ = 31.98%). Detailed forest plots of the meta-analysis for each outcome measure can be found in Supplementary File.
(2) The GRADE method indicated that the certainty level of evidence for DBP, SBP, and CRF was low. The detailed search strategies and results are presented in Supplementary Table 3.
(3) The results of the Egger regression test were as follows: for DBP, *p* = 0.2136; for SBP, *p* = 0.1563; and for CRF, *p* = 0.6828.

#### 3.4.2 Secondary outcome indicators

**Figure 4:**
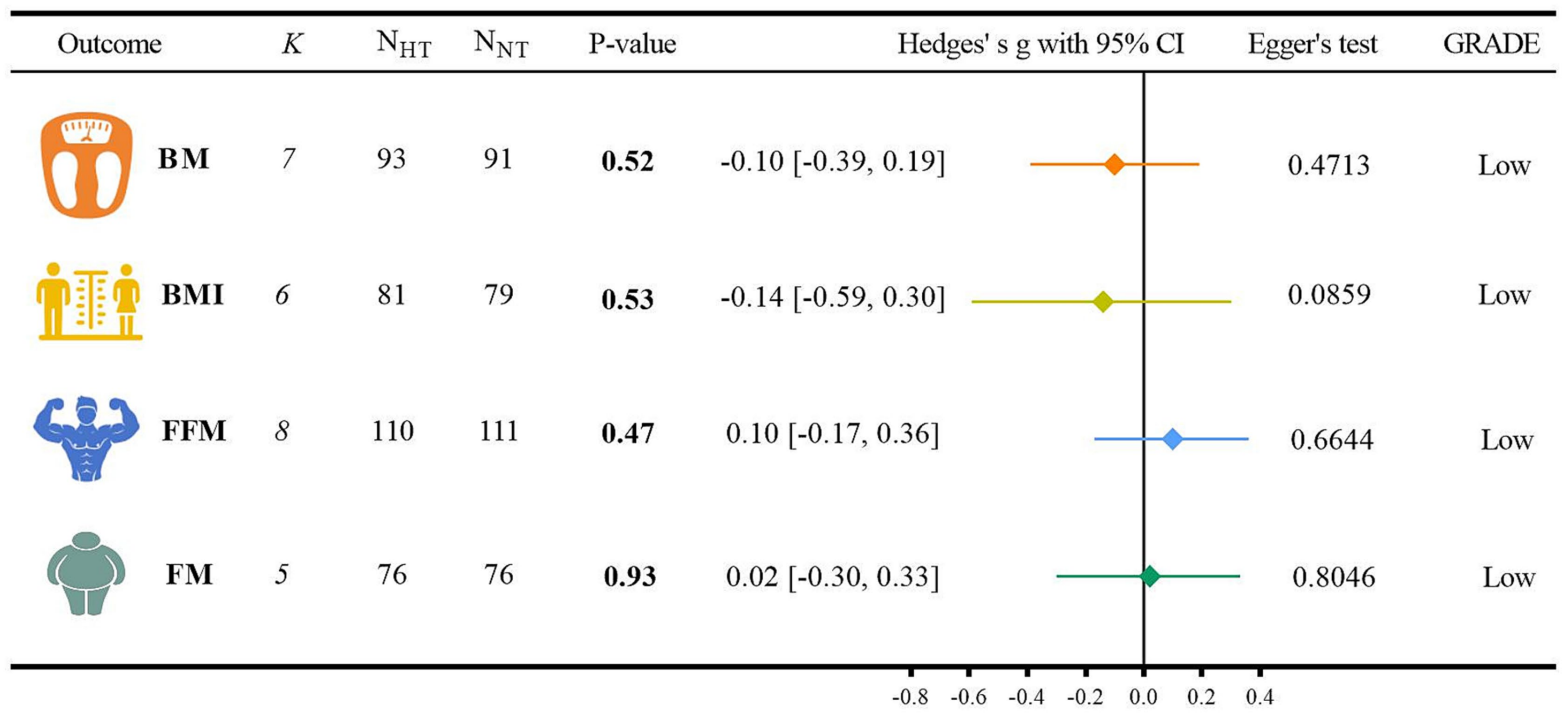
Analysis of outcome indicators of body composition.

The analysis of the outcome indicators related to body composition is shown in Figure 4.

(1) For the BM analysis, six randomized controlled trials were included (40, 47, 49, 53–55). When compared with NT, HT did not show a significant difference from NT (Hedges’ g = −0.10, 95% CI: −0.39 to 0.19; *p* = 0.52, *I*^2^ = 0.00%). For the BMI analysis, five randomized controlled trials were incorporated (40, 47, 49, 53, 55). In comparison with NT, there was no significant difference between HT and NT (Hedges’ g = −0.14, 95% CI: −0.59 to 0.30; *p* = 0.53, *I*^2^ = 49.32%). Regarding the FFM analysis, seven randomized controlled trials were involved (49, 51, 53–57). When contrasted with NT, HT did not exhibit a significant difference from NT (Hedges’ g = 0.10, 95% CI: −0.17 to 0.36; *p* = 0.47, *I*^2^ = 0.00%). For the FM analysis, four randomized controlled trials were included (51, 53, 55, 56). Compared with NT, there was no significant difference between HT and NT (Hedges’ g = 0.02, 95% CI: −0.30 to 0.33; *p* = 0.93, *I*^2^ = 0.00%). Detailed forest plots of the meta-analysis for each outcome measure can be found in Supplementary File.
(2) The GRADE method indicated that the certainty level of evidence for BM, BMI, FFM, and FM was low. The detailed search strategies and results are presented in Supplementary Table 3.
(3) The results of the Egger regression test were as follows: for BM, *p* = 0.4713; for BMI, *p* = 0.0859; for FFM, *p* = 0.6644; and for FM, *p* = 0.8046.

### 3.5 Subgroup analysis

**Figure 5:**
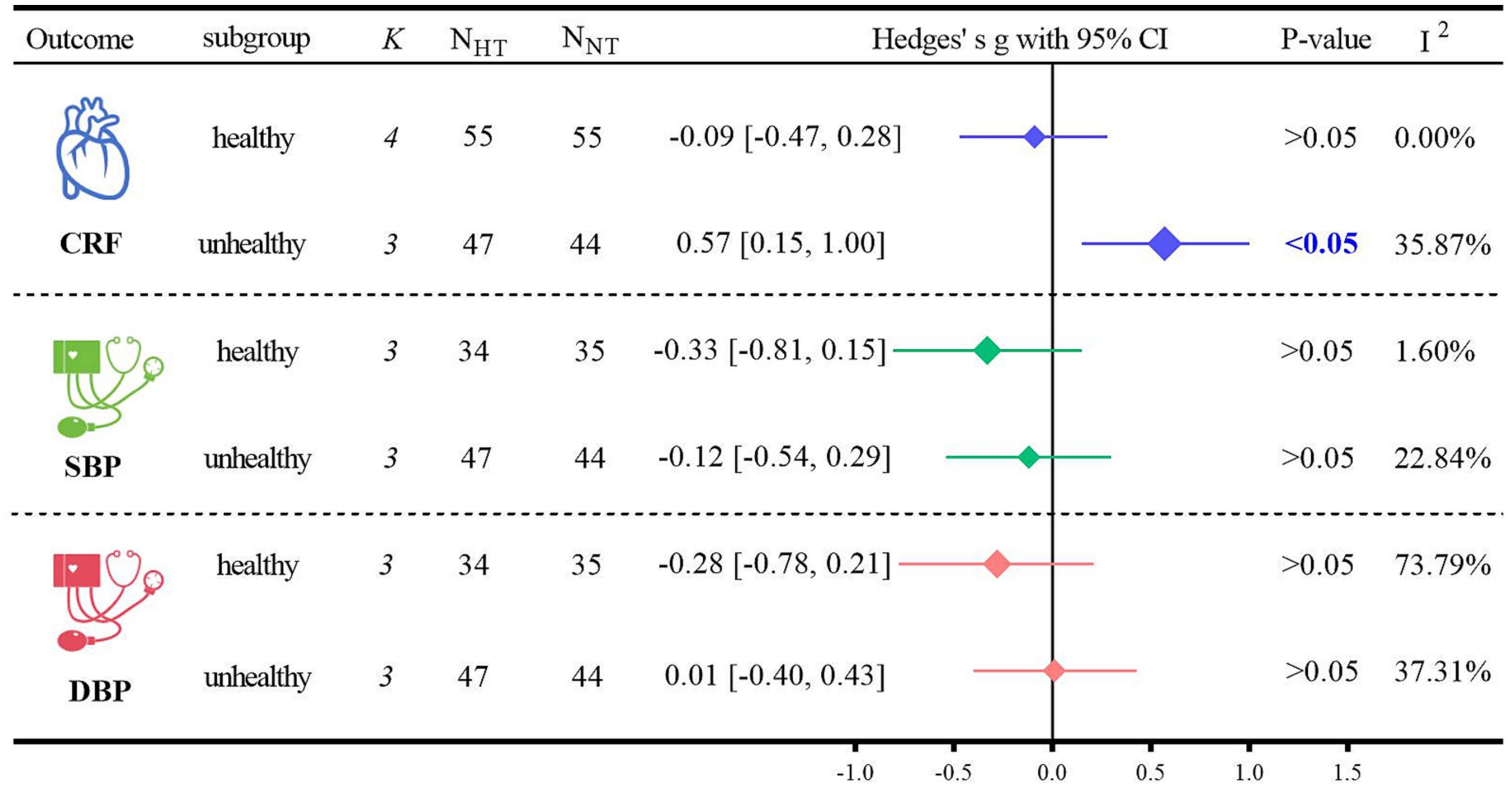
Subgroup analysis of health status.

Subgroup analyses of the primary outcome indicators were conducted based on the health status of the participants (Figure 5). Regarding CRF, HT was significantly more beneficial than NT for non-healthy older adults (Hedges’ g = 0.57, 95% CI: 0.15 to 1.00; *p <* 0.05, *I*^2^ = 35.87%). However, when comparing healthy and non-healthy older adults, there were no significant differences in blood pressure between those who received HT. For SBP, in healthy older adults undergoing HT, Hedges’ g was −0.33 with *p >* 0.05, and in non-healthy older adults undergoing HT, Hedges’ g was −0.12 with *p >* 0.05. For DBP, in healthy older adults undergoing HT, Hedges’ g was −0.28 with *p >* 0.05, and in non-healthy older adults undergoing HT, Hedges’ g was 0.01 with *p >* 0.05.

**Figure 6:**
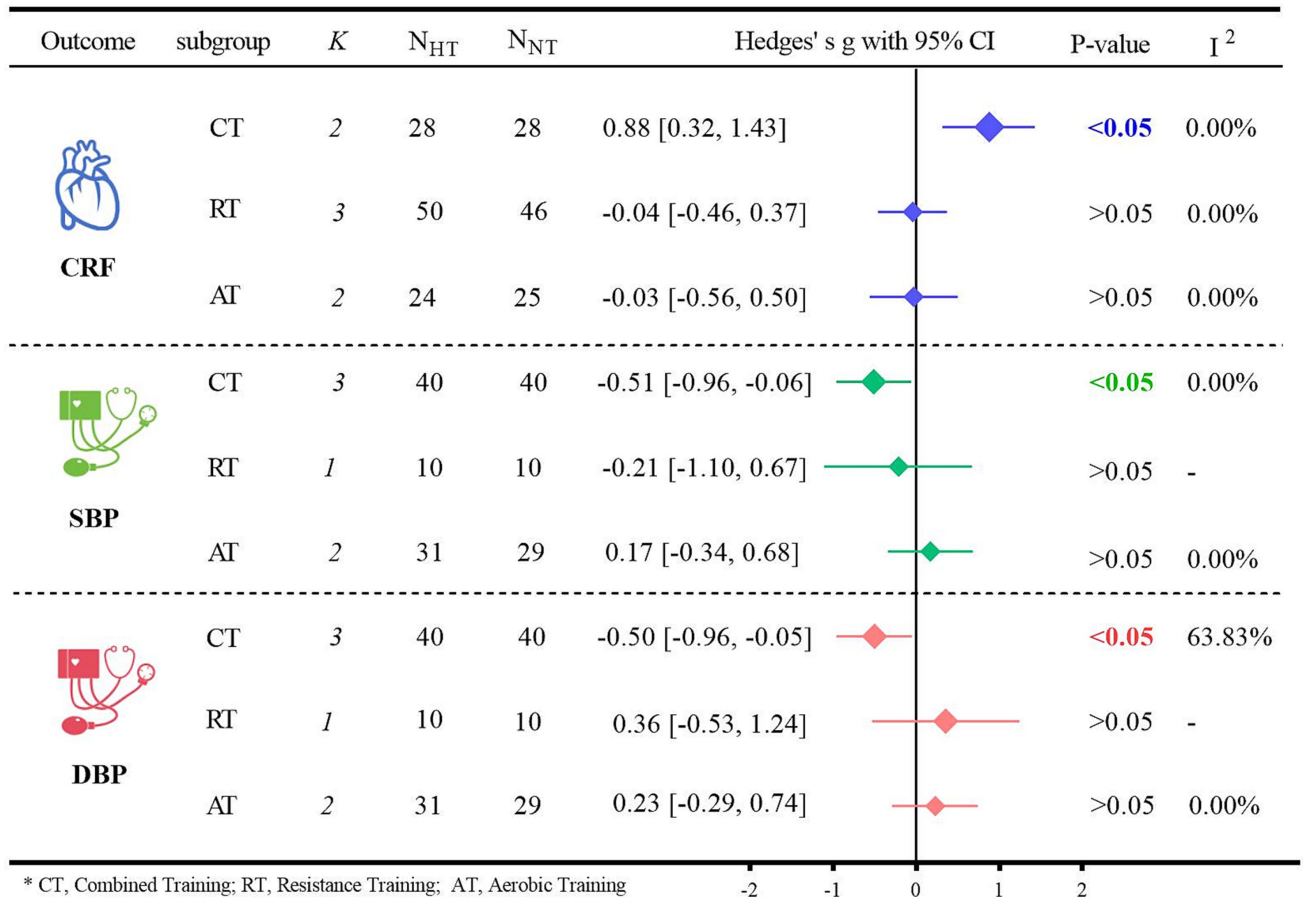
Subgroup analysis of different exercise type.

Subgroup analyses of the primary outcome indicators were conducted based on different types of exercise (Figure 6). Regarding CRF, for older adults, CT was significantly more effective than NT in terms of CRF (Hedges’ g = 0.88, 95% CI: 0.32 to 1.43; *p* < 0.05, *I*^2^ = 0.00%). However, when it came to blood pressure, neither HT nor NT resulted in a significant difference. Specifically for SBP, the results were as follows: for CT, Hedges’ g was −0.51 with *p <* 0.05; for RT, Hedges’ g was −0.21 with *p >* 0.05; and for AT, Hedges’ g was 0.17 with *p >* 0.05. For DBP, the details were: for CT, Hedges’ g was −0.50 with *p* < 0.05; for RT, Hedges’ g was 0.36 with *p >* 0.05; and for AT, Hedges’ g was 0.23 with *p >* 0.05.

## 4 Discussion

To the best of our knowledge, this is the first systematic review and meta-analysis to systematically evaluate the impact of HT on cardiometabolic health and body composition in older adults. The main finding of this meta-analysis was that HT did not have a significant advantage over NT in enhancing CRF, SBP, DBP, BM, BMI, FFM, and FM in older adults. However, subgroup analyses indicated that HT was significantly more effective than NT in improving CRF in unhealthy older adults. When the exercise modality is CT, HT can significantly enhance the CRF of older adults as well as significantly reduce their SBP and DBP.

### 4.1 Effects of hypoxic training on cardiometabolic health

Meta-analysis of this study showed that HT did not have a beneficial effect on cardiometabolic health in older adults compared to NT, as evidenced by CRF (Hedges’ g = 0.20, *p* = 0.17), SBP (Hedges’ g = −0.21, *p* = 0.19) and DBP (Hedges’ g = −0.14, *p* = 0.56). Although prior systematic reviews had proposed that HT might confer benefits to the cardiometabolic health of older adults (39), this hypothesis was not validated in the current study. Additionally, meta-analyses have demonstrated that exercise is efficacious in enhancing the cardiometabolic health of overweight or obese adults (22, 33) and that CT can have the most advantageous impact on these health indicators (58). Subgroup analyses in this study also confirmed the benefits of CT in older adults, where hypoxia combined with CT effectively increased CRF in older adults (Hedges’ g = 0.88, *p* < 0.05).

CRF serves as an independent indicator of cardiometabolic health (59). HT has been evidenced to enhance CRF in healthy adults (60). The subgroup analyses in this study further revealed that HT significantly elevated CRF in non-healthy older adults (Hedges’ g = 0.57, *p* < 0.05), indicating that HT can confer more substantial benefits on CRF in this particular subgroup of non-healthy older adults. The observed effect size (Hedges’ g = 0.57) suggests that hypoxic conditioning may confer a moderate-to-large improvement in VO_2_max and/or functional outcomes, indicating its potential clinical and practical relevance. In most previous studies, although the subjects were either obese or healthy adults, they generally did not have metabolic disorders, and their baseline markers were likely within the healthy range. This is in contrast to patients with cardiometabolic disorders, such as older adults with T2DM or those with multiple comorbidities. Exercise interventions may be more efficacious in this latter population (61). It has also been demonstrated that HT improves CRF in patients with T2DM (53, 62). This phenomenon can be attributed to the fact that in a hypoxic environment, the body has the ability to increase the production of EPO. Moreover, the dual stimulation of hypoxia and exercise can more markedly enhance the body’s physiological response and adaptive capacity to hypoxia (63). EPO has also been shown to stimulate bone formation and repair processes and to enhance cardiorespiratory fitness (64). These findings offer a novel perspective on how HT can contribute to the improvement of cardiometabolic health in unhealthy older adults.

Although the aforementioned analyses indicate that HT can confer benefits to CRF in unhealthy older adults, HT did not exhibit an advantage with respect to SBP and DBP. A prior meta-analysis also revealed that HT did not significantly improve blood pressure in overweight or obese individuals (65). Older adults may be less responsive to exercise-induced reductions in blood pressure (66), which is in line with the findings of the present study, where HT does not seem to offer additional benefits for blood pressure reduction. While medications for hypertension are effective and, in most cases, have relatively few side effects, healthcare costs are escalating (67). Exercise is well-documented to be efficacious in lowering blood pressure (68–70). The primary reason for this is that exercise suppresses the sympathetic nervous system (71), and hypertension is closely associated with overactivity of the sympathetic nervous system (72). Hypertension is a significant risk factor for CVD (73, 74), particularly among middle-aged and older adults, and it is positively correlated with an increased incidence of morbidity and mortality due to CVD (75). In a meta-analysis evaluating the effects of exercise on blood pressure, endurance training, RT, and CT were all found to reduce blood pressure (74). Moreover, a network meta-analysis confirmed that CT was the most effective exercise modality for reducing blood pressure (58). Although in the subgroup analyses of the present study, CT did significantly improve SBP (Hedges’ g = −0.51, *p* < 0.05) and DBP (Hedges’ g = −0.50, *p* < 0.05) in older adults, it did show potential advantages compared to other interventions. The proliferation of commercial devices now enables hypoxic exposure to be readily accessible by means as simple as using specialized breathing masks or staying in environmentally controlled chambers, without the need to visit high-altitude areas (76). This provides a safe, cost-effective, and highly accessible therapeutic strategy for the older adults. Consequently, incorporating increased hypoxic exposure during CT to improve CRF, SBP, and DBP in older adults may represent the optimal training modality for enhancing cardiometabolic health in this population.

### 4.2 Effects of hypoxic training on body composition

Meta-analysis of this study showed that HT did not have an effective effect on body composition in older adults compared to NT, as evidenced by BM (Hedges’ g = −0.10, *p* = 0.52), BMI (Hedges’ g = −0.14, *p* = 0.53), FFM (Hedges’ g = 0.10, *p* = 0.47) and FM (Hedges’ g = 0.02, *p* = 0.93). Existing research on the impact of HT on body composition has predominantly focused on overweight or obese populations. The findings indicate that HT is ineffective in improving body composition (65, 77, 78), which is in accordance with the results of the present study, as no potential benefits were observed in terms of enhancing body composition. Currently, there is only one study that reported that hypoxia conditioning (which involves repeated exposure to hypoxia in combination with exercise training) significantly reduced BMI in middle-aged adults. However, it did not have an effect on BMI, FFM, or FM in older adults. Nevertheless, the analyses that combined middle-aged and older adults demonstrated that, after hypoxia conditioning, both middle-aged and older adults exhibited more substantial improvements in body fat and BMI (38).

Although certain studies have evidenced that hypoxic exposure is more effective in reducing body weight and fat (41), the analyses that combined hypoxia with exercise did not reveal that HT significantly improved body composition. This outcome may be influenced by several factors. It has been shown that hypobaric hypoxia leads to an elevation in metabolic rate, a reduction in food intake, and an increase in leptin levels (41, 79). Simultaneously, when the body is exposed to hypoxia, its capacity to deliver oxygen to the muscles is enhanced, thereby increasing the efficiency of fat oxidation (80). Consequently, exposure to hypobaric hypoxia might contribute to a relatively substantial decrease in body weight and fat. Regrettably, among the literature included in this study, the exercise interventions were conducted in normobaric hypoxic environments. Thus, it remains uncertain whether such exposures to hypobaric hypoxia can yield a favorable improvement in the body composition of older adults. Moreover, formulating exercise intensity prescriptions is invariably challenging (81). A burgeoning body of research indicates that HIIT achieves remarkable fat loss, which helps to improve body composition and confers greater benefits for cardiometabolic health (82–84). Although it has been demonstrated that HIIT is safe and feasible when performed under hypoxic conditions (85), existing studies have predominantly chosen lower or moderate exercise intensities for older age groups. This could be attributed to the fact that exercise in hypoxic environments elicits a more intense metabolic response and exacerbates fatigue (86). In addition, after hypoxic exposure, the acute expression of erythropoietin is significantly higher in young individuals compared to older adults, and the return to normal levels occurs more rapidly (87). The severity of hypoxia may also be a crucial factor influencing body composition. A meta-analysis suggests that exercise at moderate altitudes (1500–3,500 m) can effectively reduce body fat (88). The hypoxia range included in the present study (FiO_2_: 16.1–14.4%) was in line with moderate altitude, yet no significant improvement in body fat was observed in older adults. However, some findings have demonstrated that the incorporation of hypoxic exposure (FiO_2_ = 17.2%) during high-intensity training can result in improvements in the body composition of obese women. Therefore, future research should focus on considering the potential of mild hypoxia (e.g., FiO_2_ = 17.2%) combined with HIIT to improve the body composition of older adults, while also taking hypobaric hypoxia into consideration.

### 4.3 Limitations

The twelve studies included in this research had small sample sizes, with each study having fewer than 30 participants. Moreover, only two of these studies were limited to a specific gender. This study did not incorporate literature regarding hypoxia exposure, and it failed to investigate whether there were differences between exposure to hypoxia alone and the combination of hypoxia exposure with exercise interventions. The limited number of data points for each outcome indicator increases the potential for bias in the subgroup analyses. For instance, the effect of RT on blood pressure was documented in only one of the papers. The severity of hypoxia in all the included studies fell within the moderate altitude range. Additionally, this paper did not further elaborate or make more detailed differentiations within the range of hypoxia.

### 4.4 Future research directions

Further expansion of the sample size is needed to control for possible adverse effects of gender differences strictly. Additionally, specific interventions for hypoxic training tailored to the older adults population should be thoroughly investigated to determine the optimal exercise intensity and duration. In studies involving patients with T2DM, hypobaric hypoxia has been shown to reduce the risk of diabetes in both short-term and long-term interventions. Therefore, special attention should be given to exploring the benefits of combining hypobaric hypoxia with CT. In the context of studies on hypertensive patients, although no existing research has yet confirmed the benefits of hypoxic training, the present study indicates that the combination of hypoxia and combined exercise holds potential in influencing blood pressure levels in older adults. This finding may offer novel insights and recommendations for future hypoxic training programs aimed at enhancing the health of hypertensive patients. Moreover, a more in-depth exploration of the physiological mechanisms through which hypoxic training improves blood pressure is warranted. The successful translation of hypoxic conditioning into practice depends on its feasibility, safety, and accessibility in target settings. To this end, future RCTs require methodological refinements. These should include larger sample sizes to ensure sufficient statistical power, proactive investigation of sex as a biological variable, and precise definition of the therapeutic window through systematic variations in hypoxia dosage. These steps are crucial to generating clinically applicable and broadly generalizable evidence.

## 5 Conclusion

Compared with exercise in a normoxic environment, CT (aerobic and resistance training) in a hypoxic environment better improves cardiometabolic health in older adults. Moreover, hypoxic training can enhance CRF in older adults with multiple comorbidities or diabetes, playing a role in preventing and improving cardiometabolic health in this population. Limited by the number of research documents available, the above conclusions still await verification by more high-quality studies.

## Data Availability

The original contributions presented in the study are included in the article/Supplementary material, further inquiries can be directed to the corresponding author.
