# Supplementary material for: Combined training in hypoxic environments improves cardiometabolic health in older adults: a systematic review and meta-analysis of randomized controlled trials
Source: Front Med (Lausanne). 2025 Dec 3;12:1728637. doi: 10.3389/fmed.2025.1728637 (PMC12708552; doi:10.3389/fmed.2025.1728637)
Supplement: Supplementary file 1 [file Data_Sheet_1.doc]

**
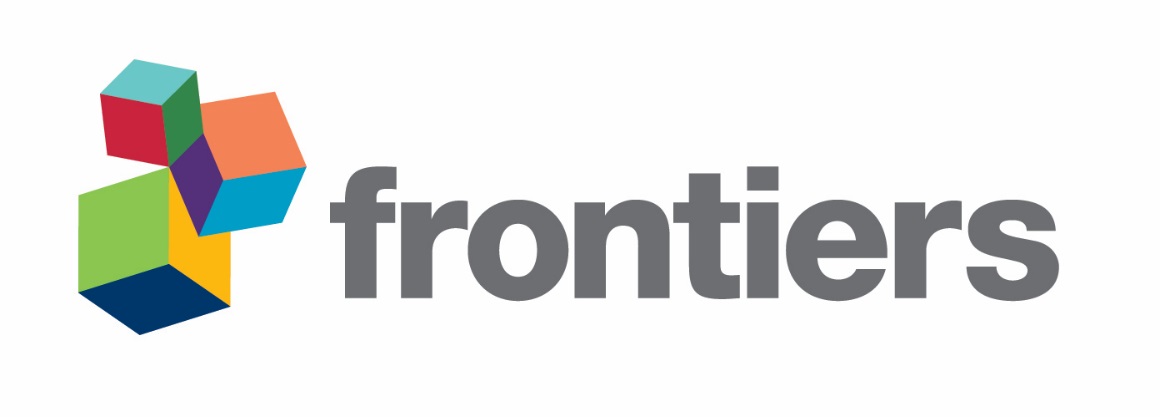
**

Supplementary Material

# Combined Training in Hypoxic Environments Improves Cardiometabolic Health in Older Adults: A Systematic Review and Meta-Analysis of Randomized Controlled Trials

[Supplementary Table 1 Search strategy 2](#__RefHeading___Toc8694)

[Supplementary Table 2. Sensitivity analysis. 4](#__RefHeading___Toc5414)

[Supplementary Table 3. Certainty of evidence for meta-analysed outcomes 5](#__RefHeading___Toc5628)

# Supplementary Table 1 Search strategy

| Databases | Search strategy | Results |
| --- | --- | --- |
| Pub Med | ((((((((Intermittent hypoxia) OR (hypoxia exercise)) OR (hypoxic training)) OR (altitude training)) OR (normobaric hypoxia training)) OR (Live high-train high)) OR (Live high-train low)) OR (Live low-train high)) AND ((((aged) OR (older adult)) OR (elderly)) OR (old people)) | 3441 |
| Web of science | (TS=(Intermittent hypoxia) OR TS=(hypoxia exercise) OR TS=(hypoxic training) OR TS=(altitude training) OR TS=(normobaric hypoxia training) OR TS=(Live high-train high) OR TS=(Live high-train low) OR TS=(Live low-train high)) ((((TS=(aged)) OR TS=(older adult)) OR TS=(elderly)) OR TS=(old people)) (#1 AND #2) | 6429 |
| EMBASE | 'intermittent hypoxia'/exp OR 'intermittent hypoxia' OR (intermittent AND ('hypoxia'/exp OR hypoxia)) OR 'hypoxia exercise' OR (('hypoxia'/exp OR hypoxia) AND ('exercise'/exp OR exercise)) OR 'hypoxic training'/exp OR 'hypoxic training' OR (hypoxic AND ('training'/exp OR training)) OR 'altitude training' OR (('altitude'/exp OR altitude) AND ('training'/exp OR training)) OR 'normobaric hypoxia training' OR (normobaric AND ('hypoxia'/exp OR hypoxia) AND ('training'/exp OR training)) OR 'live high-train high' OR (live AND 'high train' AND high) OR 'live high-train low' OR (live AND 'high train' AND low) OR 'live low-train high' OR (live AND 'low train' AND high)"elderly'/exp OR elderly OR (older AND adults)  'aged'/exp OR aged OR 'older adult'/exp OR 'older adult' OR (older AND ('adult'/exp OR adult)) OR 'elderly'/exp OR elderly OR 'old people' OR (old AND people)  #1 AND #2 | 2421 |
| Scopus | ( ( TITLE-ABS-KEY ( intermittent AND hypoxia ) OR TITLE-ABS-KEY ( hypoxia AND exercise ) OR TITLE-ABS-KEY ( hypoxic AND training ) OR TITLE-ABS-KEY ( altitude AND training ) OR TITLE-ABS-KEY ( normobaric AND hypoxia AND training ) OR TITLE-ABS-KEY ( live AND high-train AND high ) OR TITLE-ABS-KEY ( live AND high-train AND low ) OR TITLE-ABS-KEY ( live AND low-train AND high ) ) ) AND ( ( TITLE-ABS-KEY ( aged ) OR TITLE-ABS-KEY ( older AND adul ) OR TITLE-ABS-KEY ( elderly ) OR TITLE-ABS-KEY ( old AND people ) ) ) | 2194 |
| Cochrane Library | (Intermittent hypoxia):ti,ab,kw OR (hypoxia exercise):ti,ab,kw OR (hypoxic training):ti,ab,kw OR (altitude training):ti,ab,kw OR (normobaric hypoxia training):ti,ab,kw  (aged):ti,ab,kw OR (older adult):ti,ab,kw OR (elderly):ti,ab,kw OR (old people):ti,ab,kw  #1 AND #2 | 503 |

# Supplementary Table 2. Sensitivity analysis.

| Outcome | Correlation coefficient | Random effects model | | z | p-value | Tau^2^ | I^2^ (%) | Test of heterogeneity | | |
| --- | --- | --- | --- | --- | --- | --- | --- | --- | --- | --- |
|  |  | Hedge’ g | 95%-CI |  |  |  |  | Q | d.f | p-value |
| Cardiac metabolic health | r = 0.5 | -0.04 | [-0.26, 0.19] | -0.31 | 0.76 | 0.10 | 39.06 | 29.54 | 18 | 0.04 |
|  | r = 0.6 | -0.04 | [-0.29, 0.21] | -0.34 | 0.74 | 0.15 | 49.31 | 35.51 | 18 | 0.74 |
|  | r = 0.7 | -0.05 | [-0.33, 0.23] | -0.37 | 0.71 | 0.23 | 59.58 | 44.53 | 18 | 0.00 |
|  | r = 0.8 | -0.07 | [-0.40, 0.26] | -0.41 | 0.68 | 0.37 | 69.94 | 59.89 | 18 | 0.00 |
|  | r = 0.9 | -0.10 | [-0.52, 0.31] | -0.49 | 0.63 | 0.68 | 80.56 | 92.57 | 18 | 0.00 |
| Body composition | r = 0.5 | -0.02 | [-0.16, 0.13] | -0.20 | 0.84 | 0.00 | 0.00 | 14.53 | 25 | 0.95 |
|  | r = 0.6 | -0.01 | [-0.16, 0.13] | -0.19 | 0.85 | 0.00 | 0.00 | 17.26 | 25 | 0.87 |
|  | r = 0.7 | -0.01 | [-0.16, 0.14] | -0.15 | 0.88 | 0.00 | 0.00 | 21.32 | 25 | 0.67 |
|  | r = 0.8 | -0.01 | [-0.17, 0.15] | -0.11 | 0.91 | 0.02 | 11.14 | 28.14 | 25 | 0.30 |
|  | r = 0.9 | -0.01 | [-0.20, 0.19] | -0.05 | 0.96 | 0.11 | 41.90 | 43.03 | 25 | 0.01 |

# Supplementary Table 3. Certainty of evidence for meta-analysed outcomes

| Outcome | No of Participants and Studies | Gradeassessment | | | | | Certainty of evidence |
| --- | --- | --- | --- | --- | --- | --- | --- |
|  |  | Risk of bias | Inconsistency | Indirectness | Imprecision | Publication bias |  |
| BM | 156 (6 RCTs) | Serious^a^ | Not serious | Not serious | Serious^d^ | Not serious | Low |
| BMI | 132 (5 RCTs) | Serious^a^ | Not serious | Not serious | Serious^cd^ | Not serious | Low |
| FFM | 193 (7 RCTs) | Serious^a^ | Not serious | Not serious | Serious^d^ | Not serious | Low |
| FM | 124 (4 RCTs) | Serious^a^ | Not serious | Not serious | Serious^d^ | Not serious | Low |
| SBP | 132 (5 RCTs) | Serious^a^ | Not serious | Not serious | Serious^d^ | Not serious | Low |
| DBP | 132 (5 RCTs) | Serious^a^ | Not serious | Not serious | Serious^cd^ | Not serious | Low |
| CRF | 173 (6 RCTs) | Serious^a^ | Not serious | Not serious | Serious^d^ | Not serious | Low |

a: High risk of bias with allocation concealment.

b: High heterogeneity.

c: The confidence intervals indicated the potential for import harm or benefit.

d: Sample size not more than 400.


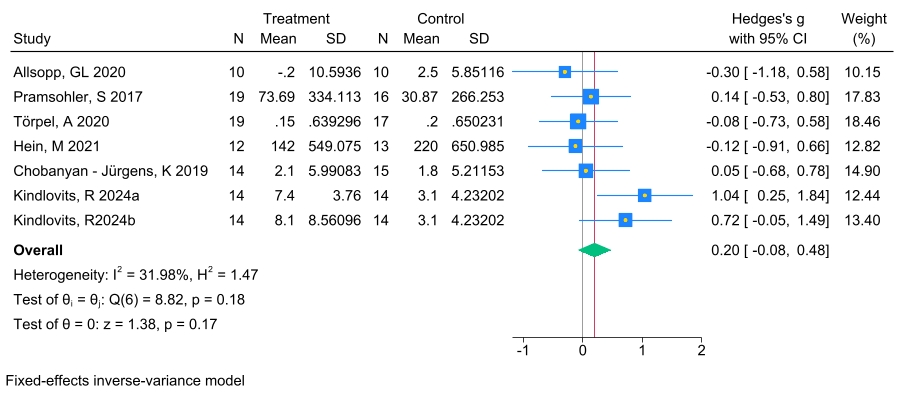


The results for CRF


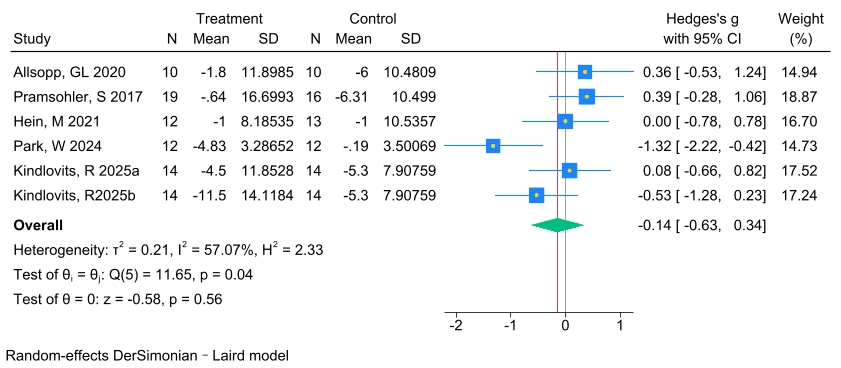


The results for DBP


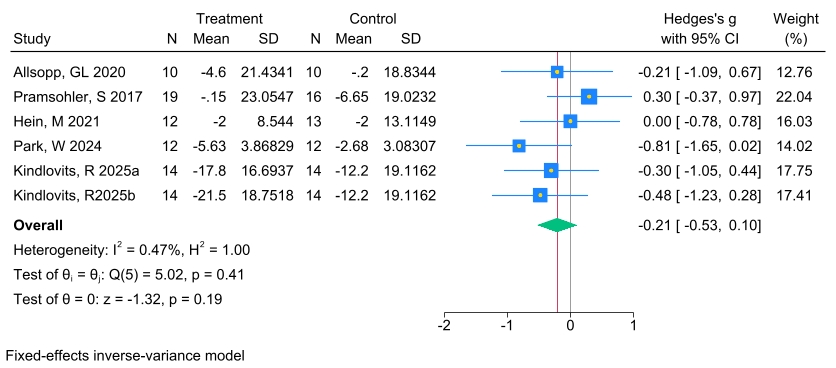


The results for SBP


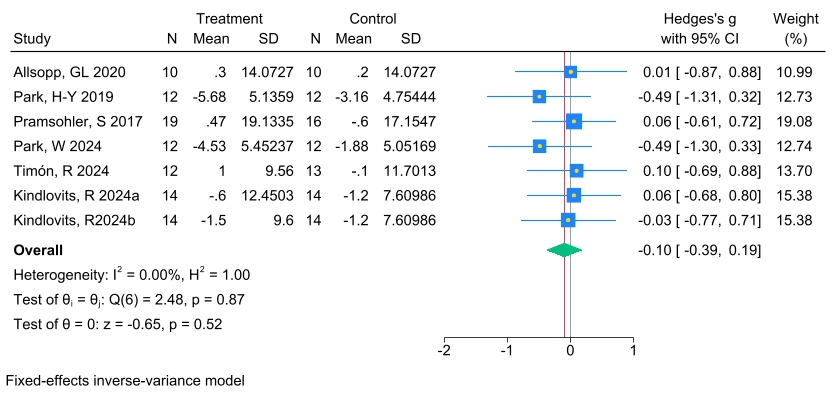


The results for BM


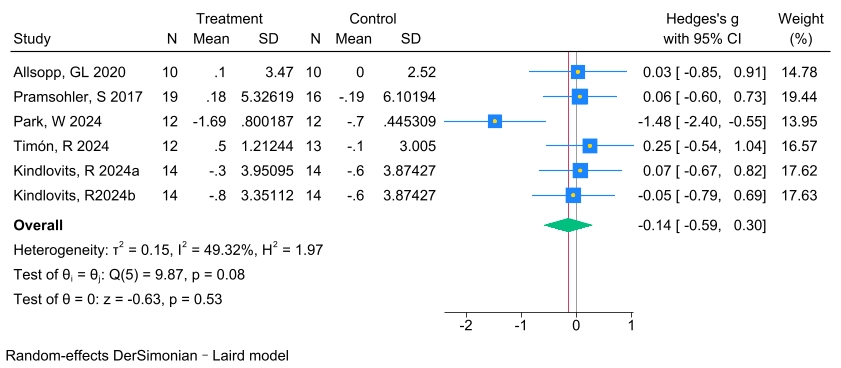


The results for BMI


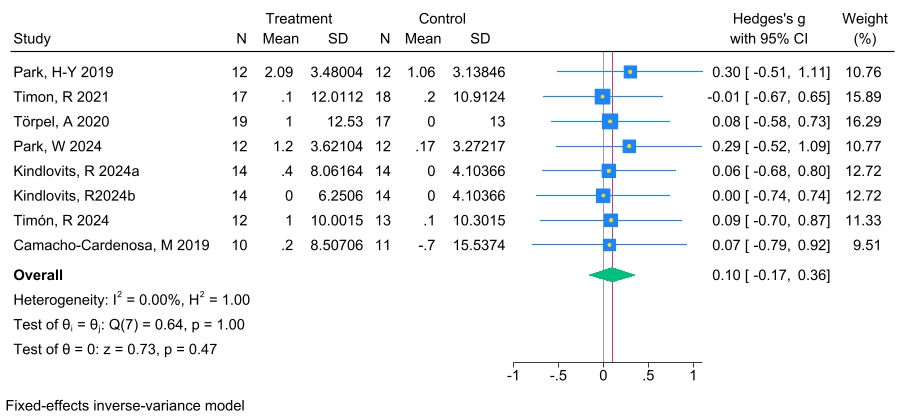


The results for FFM


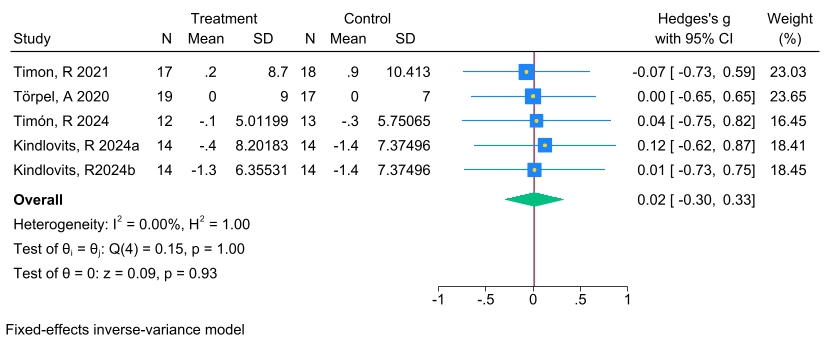


The results for FM
